# Elastic Tubing Resistance Training and Autonomic Modulation in Subjects with Chronic Obstructive Pulmonary Disease

**DOI:** 10.1155/2018/9573630

**Published:** 2018-05-29

**Authors:** Ana Laura Ricci-Vitor, Luiz Carlos M. Vanderlei, Carlos Marcelo Pastre, Dionei Ramos, Ercy Mara C. Ramos, Celso Ferreira Filho, Celso Ferreira

**Affiliations:** ^1^Department of Medicine, Federal University of São Paulo, São Paulo, SP, Brazil; ^2^Department of Physical Therapy, São Paulo State University, Presidente Prudente, SP, Brazil; ^3^Department of Clinical Medicine, Federal University of São Paulo, São Paulo, SP, Brazil

## Abstract

This study addresses evidence concerning elastic tubing resistance training (ET) on autonomic modulation in patients with chronic obstructive pulmonary disease (COPD). Autonomic dysfunction is common in COPD and contributes to the development of arrhythmias and sudden death. Along with autonomic dysfunction, muscle dysfunction is related to functional limitations and prognosis of the disease. This study investigated the effects of ET on autonomic modulation, muscle strength, and walking distance in COPD. Subjects were divided into two groups, ET (*n* = 20; 66,5 ± 8,9 y; 25,5 ± 3,5 kg/m^2^; FEV_1_/FVC: 50,3 ± 11,0) and conventional training (*n* = 19; 66,0 ± 6,9; 27,1 ± 4,3; FEV_1_/FVC: 55,05 ± 9,56). Both groups undertook 24 sessions for 60 minutes, 3 times in a week. The significance level was *p* ≤ 0,05. Autonomic modulation was evaluated using heart rate variability in the time (rMSSD, ms) and frequency domain (HF, ms). Strength for upper and lower limbs was measured using dynamometry and walking distance was measured using a 6-minute walking test. There were no significant differences in the outcomes between groups. There was an increment to rMSSD [(16,7 ± 11,0 versus 20,8 ± 14,9) versus (14,2 ± 10,0 versus 17,4 ± 12,1)], HF [(141,9 ± 191,3 versus 234,9 ± 335,7) versus (94,1 ± 123,5 versus 177,6 ± 275,5)], shoulder abduction [(50,1 ± 19,6 versus 56,9 ± 20,4) versus (50,5 ± 19,0 versus 56,9 ± 19,3)], knee flexion [(101,9 ± 34,0 versus 116,8 ± 43,3) versus (98,6 ± 21,5 versus 115,1 ± 30,8)], and walking test [(433,0 ± 84,8 versus 468,9 ± 90,8) versus (397,4 ± 99,8 versus 426,3 ± 101,6)] after training for ET and conventional training, respectively. In conclusion, ET improves autonomic modulation in COPD with additional benefits for strength and cardiorespiratory capacity similar to conventional training.

## 1. Introduction

Subjects with chronic obstructive pulmonary disease (COPD) usually present several comorbidities [1, 2], among which muscle dysfunction stands out along with impact on functional limitations and in the prognosis of COPD [1]. In addition, autonomic impairment contributes to the development of cardiac arrhythmias and sudden death [2]. Although some disease characteristics may be alleviated with drug therapy [3], muscle strength requires complementary approaches for treatment [4].

It has recently been recognized that conventional isolated resistance training (CT) performed with pulley equipment improves autonomic modulation in COPD [5–7]. In addition, we know that resistance exercise using elastic tubing improves strength in the elderly [8] and it can improve walking distance and muscle strength in COPD [9]. However, there is no evidence concerning the efficacy of elastic tubing (ET) resistance training on the outcome of autonomic modulation.

Resistance training with elastic tubing enables smoother load progression compared to conventional training and is recommended as a safe and effective strategy for enhancing muscle strength and walking distance [8, 9], presenting high acceptance, requiring minimum space, and entailing comparatively low costs [8, 9]. Taking these aspects into account, as well as the importance of collecting the frequently scarce evidence on alternative forms of exercise [10], we hypothesized that resistance training with elastic tubing would be an effective option for the treatment of autonomic impairment in COPD and that it would also improve muscle strength and walking distance.

Thus, this study aimed to investigate ET effects on autonomic modulation, muscle strength, and walking distance in COPD in comparison with CT.

## 2. Methods

### 2.1. Study Design

This trial was designed to assess the effects of 24 exercise sessions on a group of subjects with COPD assigned to resistance training using ET. The effects were compared with a group assigned to CT, looking at the outcomes of autonomic modulation, walking distance, and peripheral muscle strength. The initial evaluations were performed before the exercise sessions. The exercise sessions were held 3 times a week over 8 weeks. The final evaluations were performed up to one week after to finish the last exercise session.

### 2.2. Study Subjects

The subjects for the study were recruited in* Presidente Prudente, São Paulo,* Brazil, through referrals from a pulmonologist and information distributed to local health units supporting patients with COPD. The research staff screened subjects by telephone interview to confirm eligibility based on the inclusion criteria. Those who met the prescreening criteria then underwent a face to face evaluation and baseline assessment.

Eligibility criteria included a clinical diagnosis of COPD and clinical stability, as well as willingness to be assigned to either of the interventions. The exclusion criteria were exacerbation, recent changes in drug therapy, and lack of availability during the experimental protocol. In addition, subjects who presented less than 95% sinoatrial node beats were excluded.

Baseline assessments included anamnesis, anthropometry, and spirometry. After this, subjects were allocated into the CT or ET groups.

This clinical trial protocol was approved by the Ethics Committee of the Federal University of São Paulo (CAAE: 33726314.8.0000.5505) and all subjects signed the informed consent term. In addition, all experiments were performed in accordance with the Declaration of Helsinki.

### 2.3. Test Procedures

Outcomes were measured at baseline and after 24 sessions of resistance training. Assessments and training were performed in the morning to minimize the influence of circadian rhythm in a location with a controlled temperature between 21 and 23°C and relative humidity between 40% and 60%.

Anamnesis was performed to identify the subjects and confirm eligibility [5]. Body mass and height were measured to check anthropometry [5]. Spirometry was carried out to check pulmonary function [5].

Subjects were instructed to follow their usual drug therapy routine during the intervention; however, they were instructed to avoid the consumption of stimulants such as alcoholic drinks, coffee, and tea or food containing caffeine for 12 hours prior to the assessments. Also, those taking drug therapy such as a bronchodilator, mucolytic drugs, or anti-inflammatories were asked to avoid the medication for 12 hours if they had suffered no symptoms and bring it with them to take after the evaluation. This information was controlled through self-report. The presence or absence of adverse effects was observed during the assessments and training.

### 2.4. Intervention by Training

The protocols consisted of five movements integrated into a routine with a warm-up before and a cool-down at the end of each session. The movements were designed based on previous studies [5, 9] and performed three times a week on nonconsecutive days. Training was performed in groups of up to 12 subjects and guided and supervised by four physiotherapists. The ratings of perceived exertion were monitored using the BORG Scale of Perceived Exertion during all sessions [11]. In the case of absence during the training program, the patient continued the program until 24 sessions had been completed.

#### 2.4.1. Elastic Tubing Training

Resistance training was performed using elastic tubing (Lemgruber brand, Brazil) with internal diameters of 4 mm and 6 mm and external diameters of 8 mm and 12 mm for upper and lower limbs, respectively. Initially, the variability of individual repetitions was assessed through the fatigue resistance test that aims to establish the highest number of repetitions performed between 40 and 60 seconds before failure. Failure was considered as a decrease in range of motion or speed of motion or muscle compensation. To carry out this test, subjects were instructed to perform the maximum number of repetitions through the higher range of motion with a maximum speed, free of signs and symptoms. The test was stopped when the subject experienced fatigue. A new test was performed when the time was lower or higher than the reference. The execution time and number of repetitions were recorded and used to calculate the number of repetitions for the training, which was proportional to 20 seconds. Two sets were performed and the volume of the exercise was increased by one set every two sessions up to seven sets. Subjects were allowed a two-minute rest interval between each set. After 12 sessions, the fatigue resistance test was repeated to determine the number of repetitions for the next 12 sessions and the intensity was progressively increased [9].

#### 2.4.2. Conventional Training

Conventional training was performed using weight lifting with pulley equipment (Ipiranga, Gym Line, Brazil). Initially, the individual maximum repetition was evaluated through the one repetition maximum (1RM) that aimed to establish the maximum load for one repetition before movement failure. Failure was defined as an incomplete range of motion or muscle compensation. To carry out the test, subjects were instructed to perform the higher range of motion, initially with 20% of body mass for lower limbs and 5% of body mass for upper limbs. After a rest of five minutes, the load was increased by 5% or according to the exertion perceived, up to five attempts. The test was stopped at failure and the maximum load was assumed as the final load lifted before failure. The maximum load was recorded and used to calculate the percentage of 1RM for the training. Three sets of 10 repetitions at 60% to 80% 1RM were performed. Subjects were allowed a two-minute rest interval between each set.

### 2.5. Primary Outcome

The primary outcome consisted of indicators of autonomic modulation which were measured by HRV and expressed through indices in the time and frequency domains. Subjects were instructed to remain silent, awake, at rest, and breathing spontaneously for 30 minutes in the supine position. After the procedures, an elastic strap was placed on the subject at the height of the xiphoid process, and a heart rate receptor was attached to the wrist (Polar Electro, model S810i or RS800, Finland). This equipment has been previously validated to record beat-to-beat heart rate and HRV analysis [12–14].

The temporal recording of the interval between consecutive cardiac beats (RR) was submitted to digital filtering using Polar software and only sets with more than 95% sinoatrial node beats were included in this study [15]. Following this, the five minutes from the beginning and end of the recording were excluded from the analysis and RR intervals were selected only from the most stable part of the recording. These intervals were entered in a spreadsheet and submitted to complementary manual filtering, in which the beats were organized in ascending order and extreme values were excluded. They were reorganized into the original sequence and 256 intervals were sent to the Kubios software to run HRV analysis (Biosignal Analysis and Medical Image Group, Department of Physics, University of Kuopio, Finland) [16].

The analysis included the rMSSD (the square root of the mean squared difference between adjacent RR intervals) and SDNN (standard deviation of all normal RR intervals) indices in the time domain. Spectral components of low frequency (LF: frequency between 0.04 and 0.15 Hz) and high frequency (HF: frequency between 0.15 to 0.4 Hz) in absolute values expressed in ms^2^ were analyzed. The LF/HF ratio as a measure of sympathovagal balance was included in the analysis. Components of very low frequency (VLF: frequency between 0 and 0.04 Hz) and total power in percentages were also evaluated. Spectral analysis was calculated using Fast Fourier Transform [17]. Physiologically, rMSSD and HF represent the parasympathetic components of the autonomic nervous system in the time and frequency domain, respectively [17]. The SDNN, as a temporal index, represents global variability, while LF index, from the frequency domain, represents the action of both autonomic components, parasympathetic and sympathetic [17]. The VLF index seems to be physiologically related to the renin-angiotensin-aldosterone system and peripheral vasomotor tonus [17].

Regarding the geometric indices, the triangular index (RRtri), triangular interpolation of RR intervals (TINN), and Poincaré plot were included (SD1, SD2, and SD1/SD2 ratio) in the analysis. The RRtri and TINN indices were calculated from the density histogram of RR intervals, which show all possible values of RR intervals on the horizontal axis and the frequency on the vertical axis. The union of the column points of the histogram forms a figure resembling a triangle; the indices were extracted from this. The RRtri consists of the integral of the histogram (the total number of the RR intervals) divided by the maximum of the density distribution (modal frequency of RR intervals), measured on a discreet scale with boxes of 7.8125 ms (1/128 seconds). The TINN consists of the width of the baseline measured as the base of the triangle [17]. The Poincaré plot consists of the plot of the RRn interval duration (iRRn) on *x*-axis against the following interval (iRRn + 1) on *y*-axis. Each point in the graph corresponds to the two consecutive beats (iRRn and iRRn + 1) and when all points are plotted, it forms a figure. Following this, an ellipse is built according to the figure distribution, and an identity line and parallel line from the identity are identified. The standard deviation of the points on the identity line describes the long-term variability (SD2) and standard deviation of the points parallel to the identity line represents the instant variability (SD1) [7]. Considering physiologically geometric indexes, RRtri, TINN and SD2 represents global variability, it means both, sympathetic and parasympathetic components of the autonomic nervous system, while SD1 represents parasympathetic branch [17].

All the indexes used above are necessary since some of them represents the same branches of the autonomic nervous system they are calculated from different forms, which is important to overlap limitations from each other and could be analyzed together.

### 2.6. Secondary Outcomes

The secondary outcomes consisted of indicators of peripheral muscle strength and walking distance which were measured, respectively, by dynamometry and the six-minute walking test (6MWT).

Muscle strength was assessed unilaterally (with the dominant limb) before and after 24 sessions of training by digital dynamometry (Force Gauge® brand, model FG-100 kg), and the results were expressed in Newton (N). To facilitate this, subjects were guided to perform the movement with resistance provided by an inextensible strap coupled to the dynamometer. One extremity of the strap was fixed to a bar and the other to the body segment performing the movement. After this, the subject was instructed to perform maximum isometric contraction for six seconds followed by release of the limb. This measure was repeated a minimum of five times with a one-minute rest interval between attempts and the highest value was recorded [5]. The same five movements of training were evaluated with regard to strength. During muscle strength measurement, subjects were placed according to the following positions.

*(i) Knee Flexion*. It is a sitting position, with hip flexion and knees at 90°. The strap was attached using an ankle adapter, and the subject was instructed to perform knee flexion against the resistance.

*(ii) Knee Extension*. It is a sitting position, with hip flexion and knees at 90°. The strap was attached using an ankle adapter, and the subject was instructed to perform knee extension against the resistance.

*(iii) Shoulder Flexion*. It is a standing position, with the shoulder at 70° and the elbow in prone position. The strap was attached to a hand grip and the subject was instructed to perform shoulder flexion against the resistance.

*(iv) Shoulder Abduction*. It is a standing position, with the shoulder at 70° and the elbow in prone position. The strap was attached to a hand grip and the subject was instructed to perform shoulder abduction against the resistance.

*(v) Elbow Flexion*. It is a standing position, with the arm attached to the lateral region of the body. The strap was attached to a hand grip and the subject was instructed to perform elbow flexion to 90° in supine position against the resistance.

Walking distance was assessed before and after the resistance exercise program through the 6MWT, according to American Thoracic Society guidelines [18]. Blood pressure, oximetry, dyspnea level (BORG Scale), heart rate, and respiratory rate were monitored during the test. Standardized verbal performance incentives were given every minute. The test was performed on a 30-meter track, free of people, and was repeated twice with an interval of 30 minutes between attempts and the highest value was taken into account [5].

### 2.7. Statistical Analysis

The profile of the population was characterized descriptively and the results were expressed in values of mean, standard deviation, median, and interquartile range or in percentage and absolute values. The difference between groups for the categorical variables at baseline was tested using the Chi-square test.

For the inferential statistics, normality was evaluated through the Shapiro-Wilk test. After this, the intergroup differences (ET and CT) were evaluated through the delta between the moments before and after the training, followed by Student's *t*-test for nonpaired data in case of the normal distribution or Mann–Whitney test in case of nonnormal distribution. The intragroup differences between the moments before and after training were evaluated through Student's *t*-test for paired data in case of the normal distribution or the Wilcoxon test in case of nonnormal distribution. Differences were considered significant at *p* ≤ 0.05.

## 3. Results

**Figure 1:**
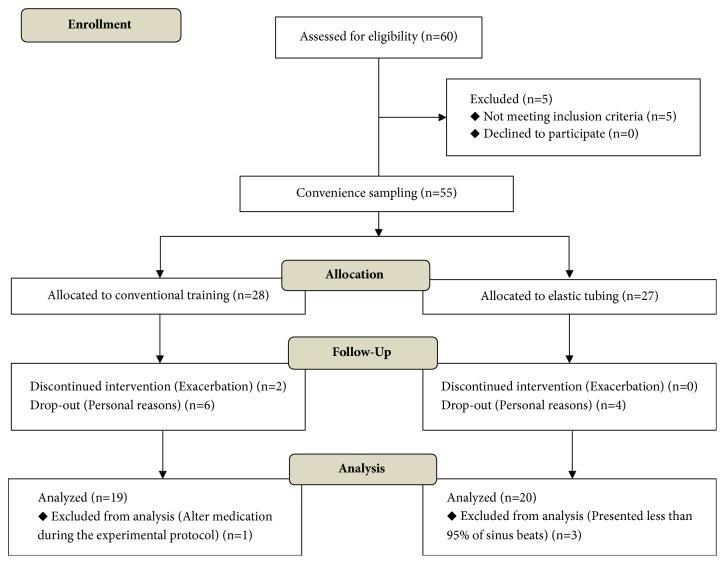
The flow diagram describes the process of the trial design since the enrollment, the follow-up, and allocation until the data analysis.

The flow diagram of the progress through the phases of the trial for the two groups (including enrolment, allocation, follow-up, and data analysis) is based on the CONSORT statement [19] and represented in Figure 1.

The initial sample characterization is described in Table 1. In addition to Table 1, the mean of age for both groups was 66 years and there was no difference between groups at rest. Considering the risk factor characterization of the volunteers, it was further observed that 78,95%, *n* = 15, versus 60,00%, *n* = 12, suffered from arterial hypertension; 5,26%, *n* = 1, versus 5,00%, *n* = 1, suffer from dyslipidemia; 5,26%, *n* = 1, versus 10,00%, *n* = 2, suffer from diabetes mellitus; and 10,53%, *n* = 2, versus 0,00%, *n* = 0, reported that they were smokers for CT versus ET groups, respectively. In addition, regarding the more frequent comorbidities, less than 20,00% (*n* = 4) of the subjects of the study present arrhythmia, anxiety/depression, thyroid disorder, and fibromyalgia.

The figures for maintenance medications were as follows: 57,89%, *n* = 11, versus 65,00%, *n* = 13, used *β*2-agonists plus corticosteroids; 42,11%, *n* = 8, versus 25,00%, *n* = 5, used anticholinergics; 15.79%, *n* = 3, versus 20,00%, *n* = 4, used methylxanthines; 15,79%, *n* = 3, versus 20,00%, *n* = 4, used *β*2-agonists; 26,32%, *n* = 5, versus 5,00%, *n* = 1, used *β*2-agonists plus anticholinergics; 0,00%, *n* = 0, versus 10,00%, *n* = 2, used corticosteroids; 31,58%, *n* = 6, versus 10,00%, *n* = 2, used an angiotensin-converting enzyme inhibitor; 21,05%, *n* = 4, versus 15,00%, *n* = 3, used calcium channel blockers; 47,37, *n* = 9, versus 25,00%, *n* = 5, used diuretics; 36,84%, *n* = 7, versus 25,00%, *n* = 5, used an angiotensin-II AT1 receptor antagonist; 15,00%, *n* = 3, versus 5,26%, *n* = 1, used inhibitor of serotonin receptor; 10,00%, *n* = 2, versus 5,26%, *n* = 1, used inhibitor of the HMG-CoA reductase; and 10,00%, *n* = 2, versus 0,00%, *n* = 0, used levothyroxine sodium for CT versus ET, respectively. In addition, regarding the more frequent medications, less than 5,26% (*n* = 1) of the subjects of the study used acetylsalicylic acid, prednisolone, digoxin, *β*-blockers, histamine antagonist, hydroxychloroquine, miosan, betamethasone dipropionate, isoflavone, metformin, and gliclazide.

### 3.1. Primary Outcomes

Concerning autonomic modulation, there are no statistical differences between the effects of ET and CT in the COPD subjects. This is represented in Table 2.

The intragroup differences (Table 3) showed an increase in parasympathetic modulation for both groups after ET and CT through a significant increase in RMSSD (ms), HF (ms^2^), and SD1 (ms). In addition, sympathetic predominance can be observed before training for both groups, which remained after training. Also a significant increase for TINN and total power, which represents global variability, was observed only for CT.

### 3.2. Secondary Outcome

Regarding functional cardiorespiratory performance and peripheral muscle strength, there were no significant differences between the training groups at rest (Table 2). There was an increase in the distance walked and an increase in peripheral muscle strength for knee flexion and shoulder abduction for both groups after training. An increase in shoulder flexion was also observed only for the CT group, as demonstrated in Table 3.

Regarding adverse effects, injury arising from friction of elastic tubing with the skin was observed. To correct the friction, the affected region was protected with a towel and tape. Also, light muscle pain arising from the training adaptation was observed for both groups. There were no severe effects such as falls, hypotension symptoms, or fainting.

## 4. Discussion

The main results of this study showed that ET was effective in improving cardiac autonomic modulation, peripheral muscle strength, and walking distance in subjects with COPD without differences in comparison with CT.

The results of this study showed an increase in temporal and spectral indices of HRV (rMSSD, SD1, and HF) after training for both groups, suggesting an increase in parasympathetic modulation; there was no statistical difference between groups.

The decrease in parasympathetic modulation in COPD is already known [20–22]. This is a negative condition as the autonomic nervous system is responsible for sinoatrial node discharge rate modulation, atrioventricular node conduction speed modulation, and ventricular contractility [23]. Thereafter, these changes could contribute to the development of cardiac arrhythmias and sudden death [24, 25]. A decrease in HRV is related to an increase in morbidity and mortality in several conditions [26–29]. Both training modalities were able to promote parasympathetic increase in autonomic modulation of the evaluated subjects, suggesting benefits in COPD.

Regarding the global variability indices, the only difference was presented in the TINN index for the CT. This difference was not significant between groups. The other indices (SDNN, SD2, LF, and LF/HF) were not different, suggesting an occasional difference.

In addition, even though there are higher values of LF when compared to HF, no significant differences were found. There was also an increase in the total power components after training for CT. We suggest that the total power increment could be related to the HF and VLF increases as total power represents the sum of all frequency bands. The VLF index seems to be physiologically related to the renin-angiotensin-aldosterone system and peripheral vasomotor tonus [17]. Thus, studies evaluating the relation between chronic resistance training with those systems and the effects on long-term control of blood flow and blood pressure could be promising.

Concerning the effects of resistance training on the autonomic modulation, a recent review suggests that this does not cause important alterations in healthy subjects with normal autonomic function [30]. In this sense, this study is also relevant, as COPD is a chronic disease; it is related to autonomic impairment, and resistance training, performed with both elastic tubing and pulley equipment, could contribute to improved autonomic modulation in this population.

With respect to conventional resistance training, the literature does not present consensual results on HRV outcomes, showing changes or not in autonomic modulation according to the sample [31–35]. These differences could, at least in part, arise from the training characteristics. In COPD, only conventional resistance training with a pulley was recently recognized to increase strength and autonomic modulation [5–7]. The present study showed that ET could be used in the same way as the conventional training for this focus.

In addition to the scarcity of information with regard to resistance training and autonomic modulation, which requires investigation, increasingly, new types of training are available and demand scientific evidence [10, 27]. Elastic tubing, for example, is a cheap and portable resource, allowing more functional movements. It is currently used as rehabilitation not only for articular problems in healthy subjects but also in the elderly and subjects with chronic diseases [8]. In COPD, the present study brings evidence of benefits in important outcomes such as muscle strength and functional cardiorespiratory performance [9]; moreover, this is the first study to include autonomic modulation.

This study also found improvement in muscle strength and functional cardiorespiratory performance. The ET group increased distance walked with a mean of 35.83 meters compared to the CT, 28.89 meters. Zambom-Ferraresi et al. [36] found an increase in the 6MWT after conventional resistance training in COPD. Ramos et al. [9] observed an increase in this variable after CT and ET.

Concerning muscle strength, the most desirable benefit of the resistance training, we observed an increase for both upper and lower limbs for the evaluated movements, with significance for shoulder abduction and knee flexion. There was no difference between groups. Additionally, there was a significant increase in shoulder flexion only for the conventional training group.

This study has some limitations. The training programs were created from different tests. While the conventional training was prescribed from the 1MR test, the ET used the fatigue resistance test. Despite the difference, these tests have been prescribed and used previously [5, 7]; in addition, the equivalent moderate intensity was guaranteed for both training modalities through ratings of perceived exertion using BORG Scale.

Despite the limitations, ET has important clinical implications. It could be one more alternative for subjects who are resistant to CT. In addition, trained subjects could perform sessions at home, allowing the expansion of attending subjects at an affordable cost. Thus, evidence was found that elastic tubing could be an interesting option, according to the availability of resources in each environment and according to the treatment objectives. Additionally, it is important to point out that ET is more acceptable than conventional training for several elderly subjects [8] and there are no severe side effects, suggesting the safety and utility of this intervention for COPD.

In conclusion, we studied the effects of ET in subjects with COPD and found that ET improves cardiac autonomic modulation, peripheral muscle strength, and functional cardiorespiratory performance in subjects with COPD without differences in comparison with CT.

## Figures and Tables

**Table 1:** Sample description regarding the gender, age, anthropometry, and spirometry.

Descriptive	Elastic tubing (*n* = 20)	Conventional training (*n* = 19)	*p*
Gender (masculine^†^/feminine), *n* (%)	13 (65,00) 7 (35,00)	13 (68,42) 6 (31,58)	0,821
Body mass (kg)	67,70 ± 12,37 67,00 (19,35)	73,28 ± 13,07 75,60 (22,40)	0,179
Height (m^2^)	1,63 ± 0,10 1,66 (0,14)	1,64 ± 0,08 1,67 (0,09)	0,771
BMI (kg/m^2^)	25,53 ± 3,53 25,13 (7,09)	27,09 ± 4,29 26,47 (7,15)	0,224
FEV_1_/FVC	50,29 ± 10,96 50,2 (18,23)	55,05 ± 9,56 54,60 (16,10)	0,158
FEV_1_ (L)	1,22 ± 0,50 1,14 (0,42)	1,35 ± 0,40 1,30 (0,68)	0,158
FEV_1_ (%)	48,72 ± 16,97 41,45 (24,71)	50,63 ± 12,27 52,34 (16,71)	0,478
FVC (L)	2,47 ± 0,86 2,18 (1,29)	2,44 ± 0,65 2,41 (0,88)	0,728
FVC (%)	73,80 ± 20,92 69,97 (35,10)	72,60 ± 16,49 70,17 (16,96)	0,844

Kg: kilograms; m: meters; BMI: body mass index; FEV_1_: forced expiratory volume in the first second; FVC: forced vital capacity; %: percentage; L: liters. Data are presented in mean, standard deviation, median, and interquartile interval. Statistics were performed using *t*-test for nonpaired data or Mann–Whitney test. ^†^Chi-square.

**Table 2:** Intergroup differences for heart rate variability, muscle strength, and walking distance between the moments before and after training.

Descriptive	Elastic tubing (*n* = 20)	Conventional training (*n* = 19)	*p*
*Heart rate variability*			
rMSSD (ms)	2,25 (9,00)	2,80 (6,40)	0,923
SDNN (ms)	2,45 (13,75)	3,20 (12,70)	0,879
LF (ms^2^)	7,50 (189,50)	27,00 (138,00)	0,478
HF (ms^2^)	16,50 (138,50)	23,00 (87,00)	0,989
LF/HF	−0,03 (1,55)	0,42 (1,45)	0,459^a^
VLF (ms)	13,50 (414,50)	89,00 (276,00)	0,496
Total power (%)	193,78 (818,00)	161,00 (337,00)	0,465
TINN (ms)	2,50 (85,00)	10,00 (85,00)	0,428
RRtri	0,90 (2,87)	0,69 (3,18)	0,857
SD1 (ms)	1,65 (6,30)	2,00 (4,50)	0,945
SD2 (ms)	2,90 (19,38)	5,50 (19,10)	0,857
*Muscle strength and walking distance*			
Knee flexion (N)	14,88 ± 28,35	16,54 ± 21,05	0,550
Knee extension (N)	11,80 ± 32,52	19,91 ± 42,99	0,510*ª*
Shoulder flexion (N)	4,42 ± 14,74	9,24 ± 13,38	0,292*ª*
Shoulder abduction (N)	6,79 ± 13,67	6,44 ± 9,60	0,929^a^
Elbow flexion (N)	0,91 ± 44,00	7,45 ± 35,07	0,550
6MWT (m)	35,83 ± 73,27	28,89 ± 30,11	0,700^a^

*p*: significance level by Mann–Whitney test or “a” for Student's *t*-test for unpaired data; ms: milliseconds; ms^2^: squared milliseconds; rMSSD: the root mean square of successive difference between consecutive heart beats; SDNN: the standard deviation of normal-to-normal intervals; LF: low frequency; HF: high frequency; VLF: very low frequency; TINN: triangular interpolation of the interval between consecutive heart beats; RRtri: triangular index; SD1: standard deviation of instant variability; SD2: standard deviation of long-term interval between consecutive heart beats; 6MWT: distance walked in the six-minute walking test; N: Newton; m: meters. Values are expressed as median (interquartile range) or mean ± standard deviation.

**Table 3:** Intragroup differences for heart rate variability, muscle strength, and walking distance between the moments before and after training.

Descriptive	Elastic tubing (*n* = 20)			Conventional training (*n* = 19)		
	Before	After	*p*	Before	After	*p*
*Heart rate variability*						
rMSSD (ms)	12,65 (15,10)	19,70 (12,63)	0,015	12,50 (10,50)	16,60 (11,70)	0,040
SDNN (ms)	22,45 (20,85)	28,20 (17,55)	0,263	18,90 (12,10)	22,80 (16,90)	0,184
LF (ms^2^)	116,00 (309,00)	182,00 (349,00)	0,575	65,00 (83,00)	150,00 (264,00)	0,099
HF (ms^2^)	75,00 (187,00)	160,00 (228,50)	0,021	30,00 (121,00)	54,00 (149,00)	0,024
LF/HF ratio	1,41 (1,88)	1,63 (1,99)	0,970	2,47 (2,44)	1,70 (3,44)	0,159
VLF (ms)	203,00 (330,00)	260,00 (443,75)	0,526	116,00 (194,00)	222,00 (503,00)	0,064
Total power (%)	500,00 (837,50)	505,00 (740,00)	0,823	240,00 (296,00)	526,00 (830,00)	0,033
TINN (ms)	87,50 (72,50)	110,00 (75,00)	0,184^b^	65,00 (70,00)	95,00 (70,00)	0,029
RRtri	6,01 (4,02)	7,22 (4,15)	0,062	5,57 (3,18)	5,69 (5,59)	0,064
SD1 (ms)	8,95 (10,75)	13,95 (8,88)	0,014	8,90 (7,40)	8,20 (8,30)	0,036
SD2 (ms)	30,60 (27,53)	37,55 (23,55)	0,390	25,50 (16,50)	29,60 (23,90)	0,191
SD1/SD2	0,34 (0,13)	0,37 (0,21)	0,681	0,30 (0,15)	0,33 (0,17)	0,857^b^
*Muscle strength and walking distance*						
Knee flexion (N)	101,91 ± 34,00	116,79 ± 43,31	0,030^b^	98,58 ± 21,46	115,13 ± 30,80	0,003^b^
Knee extension (N)	197,18 ± 75,64	208,98 ± 78,44	0,121^b^	194,10 ± 55,50	214,00 ± 53,58	0,059^b^
Shoulder flexion (N)	59,60 ± 19,59	64,01 ± 23,72	0,196^b^	52,57 ± 19,81	61,81 ± 19,38	0,008^b^
Shoulder abduction (N)	50,11 ± 19,62	56,89 ± 20,37	0,039^b^	50,51 ± 18,99	56,94 ± 19,33	0,009^b^
Elbow flexion (N)	106,24 ± 60,56	107,15 ± 55,91	0,135	109,35 ± 42,08	116,80 ± 42,81	0,367^b^
6MWT (m)	433,03 ± 84,77	468,85 ± 90,79	0,041^b^	397,37 ± 99,83	426,26 ± 101,58	0,001^b^

*p*: significance level by Wilcoxon test or “b” for Student's *t*-test for paired data; ms: milliseconds; ms^2^: squared milliseconds; rMSSD: the root mean square of successive difference between consecutive heart beats; SDNN: the standard deviation of normal-to-normal intervals; LF: low frequency; HF: high frequency; VLF: very low frequency; TINN: triangular interpolation of the interval between consecutive heart beats; RRtri: triangular index; SD1: standard deviation of instant variability; SD2: standard deviation of long-term interval between consecutive heart beats; 6MWT: distance walked in the six-minute walking test; N: Newton; m: meters. Values are expressed as median (interquartile range) or mean ± standard deviation.

## Data Availability

The data spreadsheet will be available to reader's access using archived dataset in ResearchGate site.
